# Differential-Evolution Control Parameter Optimization for Unmanned Aerial Vehicle Path Planning

**DOI:** 10.1371/journal.pone.0150558

**Published:** 2016-03-04

**Authors:** Kai Yit Kok, Parvathy Rajendran

**Affiliations:** School of Aerospace Engineering, Universiti Sains Malaysia, Engineering Campus, 14300 Nibong Tebal, Pulau Pinang, Malaysia; Chongqing University, CHINA

## Abstract

The differential evolution algorithm has been widely applied on unmanned aerial vehicle (UAV) path planning. At present, four random tuning parameters exist for differential evolution algorithm, namely, population size, differential weight, crossover, and generation number. These tuning parameters are required, together with user setting on path and computational cost weightage. However, the optimum settings of these tuning parameters vary according to application. Instead of trial and error, this paper presents an optimization method of differential evolution algorithm for tuning the parameters of UAV path planning. The parameters that this research focuses on are population size, differential weight, crossover, and generation number. The developed algorithm enables the user to simply define the weightage desired between the path and computational cost to converge with the minimum generation required based on user requirement. In conclusion, the proposed optimization of tuning parameters in differential evolution algorithm for UAV path planning expedites and improves the final output path and computational cost.

## Introduction

In recent years, unmanned aerial vehicles (UAVs) have received significant attention from the military and commercial industries. Moreover, many studies have been conducted on UAV development because of its wide variety of applications, including surveillance, traffic monitoring, rescue mission, and aerial photography [1–12]. Aircraft path planning is a UAV operation that aims to generate an optimum flight path from the starting coordinate to the destination location.

Various algorithms are applicable for UAV path planning. This includes graph search algorithm [13, 14], potential field based algorithm [15], probabilistic roadmap algorithm [16, 17], rapidly-exploring random trees algorithm [18, 19], Dubin curve based algorithm [20, 21] and evolutionary algorithm [22]. Each method has its own strengths and weaknesses over others. Graph search algorithms like Dijkstra, Bellman Ford and A* are algorithms with straight forward implementation. Yet, graph search algorithms are not efficient when they are used in large search space environment.

Hence, Differential evolution (DE) algorithm has often been used in UAV path planning because of its good performance in addressing real-world optimization problems. DE a type of evolutionary algorithm, which is a stochastic, population based optimization algorithm. Thus, it may also be used in non-differential and non-linear optimization problems. In addition, DE has good convergence properties with easy implementation into applications [23–25].

There are several control parameters in DE, namely the population size, differential weight, and crossover. Obtaining an optimum value for these control parameters in DE is difficult and requires trial and error because different applications require different optimum parameter settings. DE performance is sensitive to the control parameter setting [26]. Furthermore, tuning the control parameters is often time consuming [26, 27] and justifying the optimum performance of DE is difficult. To overcome this issue, the use of mimetic algorithm in DE has been proposed [28].

Furthermore, studies claim that dynamic control parameters in the DE perform better than constant control parameters. However, most of these parameters conduct analyses by using standard test functions and are rarely specific in a particular field [25, 27, 29, 30]. Zielinski [31] has conducted a parameter study of DE by using the power allocation problem. In addition, parameter optimization has been studied in other system such as power system using Genetic algorithm and Kalman-based methods [32–36].

Besada-Portas [24] has presented a performance comparison method by using evolutionary algorithms for UAV path planning. However, this method does not analyze the control parameters in detail. Thus, the optimum control parameter strategy of DE in UAV path planning is unclear. In this study, we thoroughly investigate the effect of the control parameters in DE for UAV path planning. Thus, the proposed DE control parameter optimization expedites and improves in obtaining the desired path and computational cost for UAV path planning.

## Methodology

### Problem Formulation

**Fig 1:**
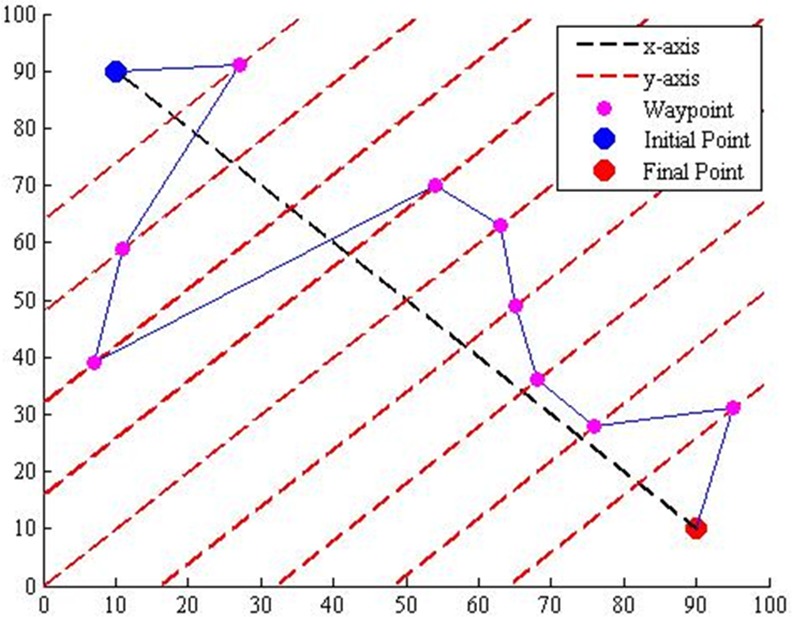
Transformation of coordinates system.

The main objective of path planning is to produce an optimum 3D flight path in both computational time and path distance from initial coordinate to target coordinate. To implement DE in path planning with high search speed, the initial point is connected with the final point as the virtual x-axis. Depending on the number of waypoint throughout the flight path, the virtual x-axis is divided into the same number of intervals and forms a virtual y-axis at each point of interval (Fig 1). The start and the goal for the desired path throughout the simulations are similar in Fig 1 which are [10, 90] and [90, 10] respectively. The map chosen for this study is from an alpine region, where the mountains and hills are treated as the path obstacles.

### Function Cost

The function costs of UAV path planning include flight path distance, real-time computational time, minimum turning radius, power consumption, and threat area. However, only the path distance and computational time are analyzed in this study. The other function costs require study using a specific UAV model to ensure impartial comparison can be done. Yet, this limits application of the proposed DE model for a particular type of UAV. Thus, by only optimizing path distance and computational time, the proposed method benefits all types of UAV.

Also, there are 5 parameters in DE algorithm, namely maximum generation number, length of solution (number of waypoint in this case study), population size, differential weight and crossover. Generally, solutions can evolve further when generation number is increased. The length of solution decides the complexity of the problem while population size, differential weight and crossover alter the performance of DE. Hence, this work analyzes the effect of population size, differential weight, and crossover on DE to obtain optimized range for those parameters.

Therefore, the UAV is assumed to carry sufficient power throughout the flight regardless of the length of the flight with no threat area on a 3D terrain map. The UAV will also maintain a minimum altitude of 100 m from the ground at each waypoint coordinate. The function cost can be evaluated by using the following:

$$J=\sum_{i=1}^{w}l_{i}\;,$$ (1)

where *w* is the number of waypoints and *l* is the length between the previous and current location.

## Principles of DE Algorithm

DE was first proposed by renowned researchers Storn and Price [37]. Similar to genetic algorithm (GA), DE involves selection, crossover, and mutation but in a different sequence. The population is initialized by randomizing individuals within a search space. The population then undergoes mutation, and an individual $v_{i}^{G+1}$ is generated by using the following equation [38–43]:

$$v_{i}^{G+1}=x_{r1}^{G}+F.(x_{r2}^{G}-\;x_{r3}^{G})\;,\;r1\neq r2\neq r3,\;i=1,2,\ldots \;,NP,$$ (2)

where *x* is an individual from the population *r ϵ* (1, *NP*), *G* is the generation or iteration, *NP* is the population size, and *F* is the differential weight. However, not all particles from the mutation will be used in the next operation, depending on crossover probability. Population with trial individuals *u*^*G*+1^ is produced by the crossover process with the following condition:

$$u_{ij}^{G+1}=\{\begin{array}{l} v_{ij}^{G+1}\;,\;rand_{ij}\leq crossover\\ x_{ij}^{G}\;,\;otherwise \end{array}\;j=1,2,\ldots \;,\;D,$$ (3)

where *rand*_*ij*_ is a random value between zero and one for the *i*^*th*^ individual at the *j*^*th*^ particle. Thereafter, the trial population will go through the selection process. Unlike GA, the selection process of DE compares the current population and trial population. The individual with lowest cost in the trial population will replace the individual of the current population:

$$x_{i}^{G+1}=\{\begin{array}{l} u_{i}^{G+1},\;f(u_{i}^{G+1})\leq f(x_{i}^{G})\\ x_{i}^{G},\;otherwise \end{array}\;.$$ (4)

The process is repeated from mutation to selection until the termination condition is met.

**Fig 2:**
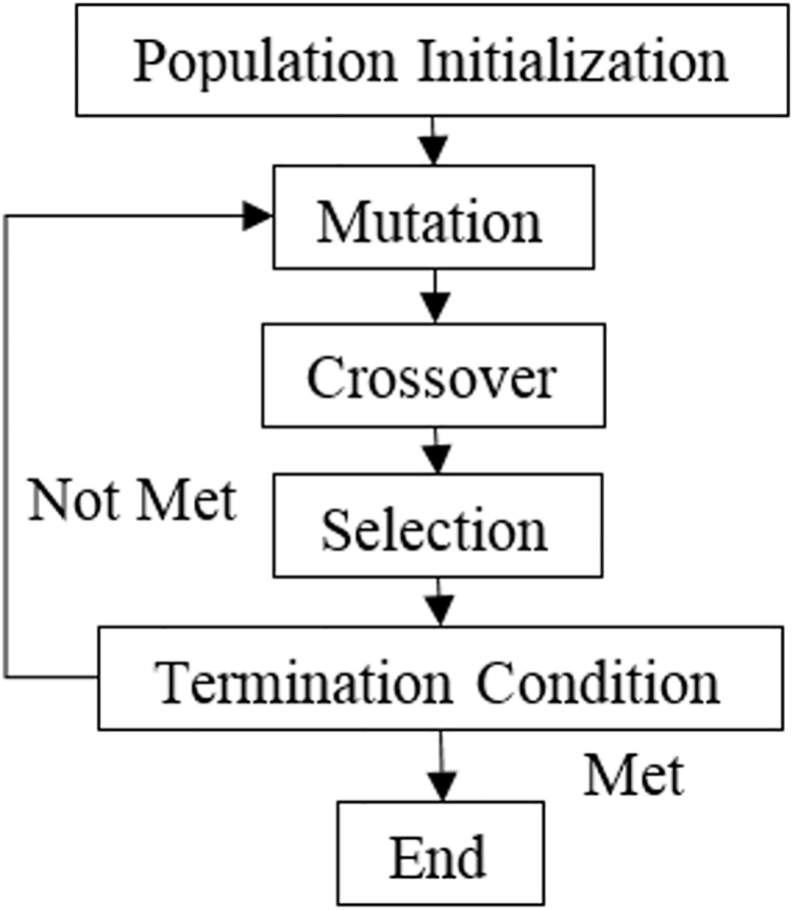
DE flowchart.

Fig 2 demonstrates the flow of the DE algorithm.

## Results and Discussions

To determine the effect of population size, differential weight, and crossover in DE, all combinations of control parameter values are tested. The range of differential weight is set from 0.1 to 1. Both crossover and population size parameters are set from 10 to 100. The generation number is from 100 to 1000. Furthermore, the number of waypoints is fixed at 50 in this investigation.

However, similar to other evolutionary algorithms, DE output depends on certain random probability to obtain better solutions; as a result, the output of DE is uncertain every time. Therefore, each combination simulation will be repeated 100 times to obtain the average output performance of DE.

**Fig 3:**
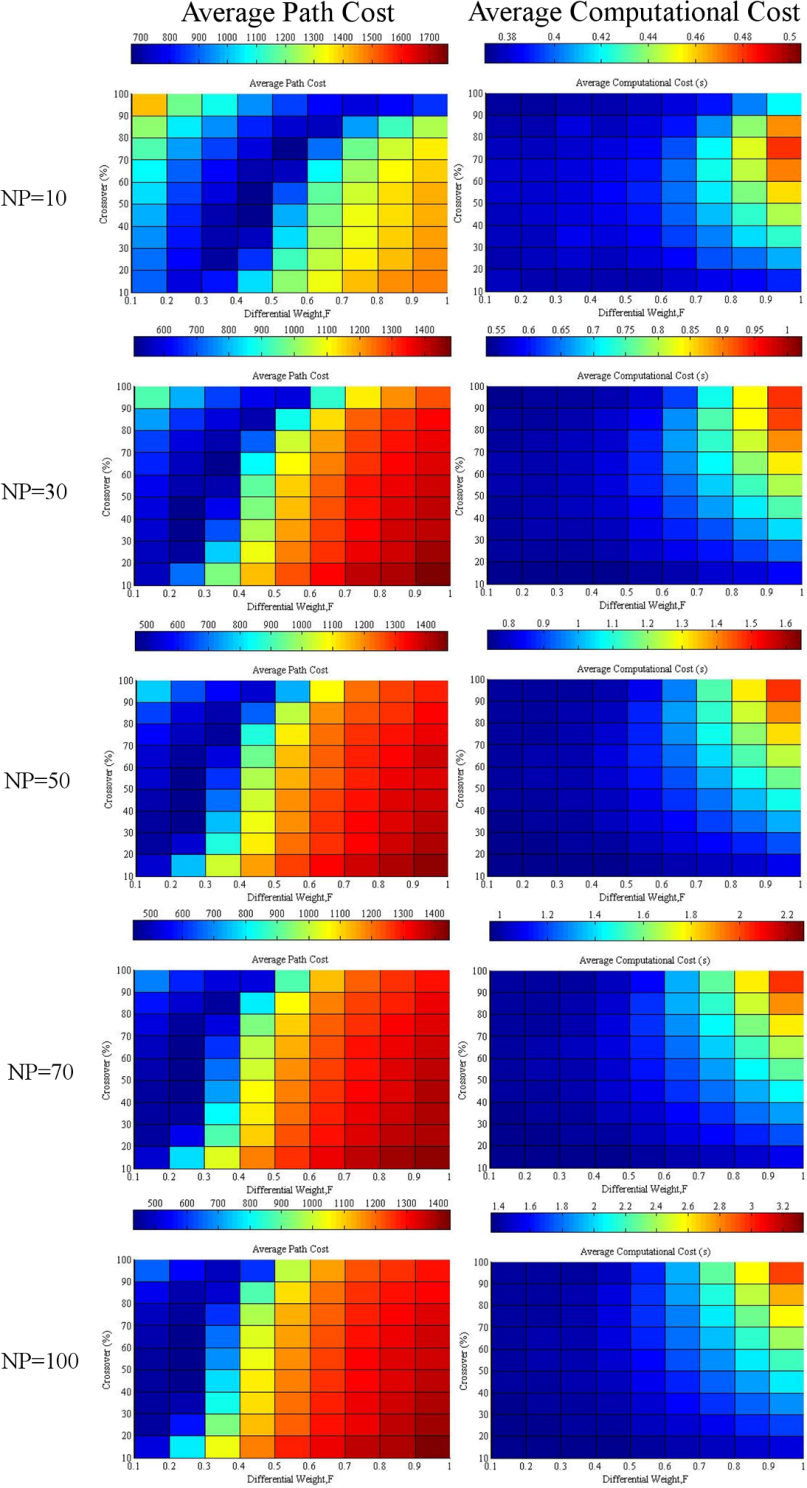
Average path and computational cost at the 1000^th^ generation with variation between differential weight and crossover at various population sizes of 10, 30, 50, 70, and 100 “Table A in S1 Dataset.”

The average path and computational cost at the 1000^th^ generation with variations in differential weight and crossover at various NPs are given in Fig 3. Increasing the differential weight will favor the reduction of the average path cost when the population size is small because it increases the diversity of the population. However, an immoderate differential weight will reduce the performance of DE.

Moreover, a bigger population size tends to limit the optimum range of differential weight because the diversity of the population is sufficient for bigger populations. An excessive differential weight will retard the decline of the average path cost. If the crossover increases, the optimum differential weight prone will increase, particularly when the population size is small.

Additionally, increasing both the crossover and differential weight increases the average computational cost. The reason behind this effect is that more work is needed for higher crossover probability, whereas a high differential weight is prone to have values outside the search space. Furthermore, extra work is required to adjust the values within the search space.

### Optimization of Differential Weight and Crossover

According to the average path and computational cost given in Fig 3, the balanced value of the differential weight at various crossover settings can be assumed at the interception point of the average path cost and computational cost. This assumption is valid because increasing the differential weight will reduce the average path cost to a certain level before it increases again with a significant increase in computational cost.

**Fig 4:**
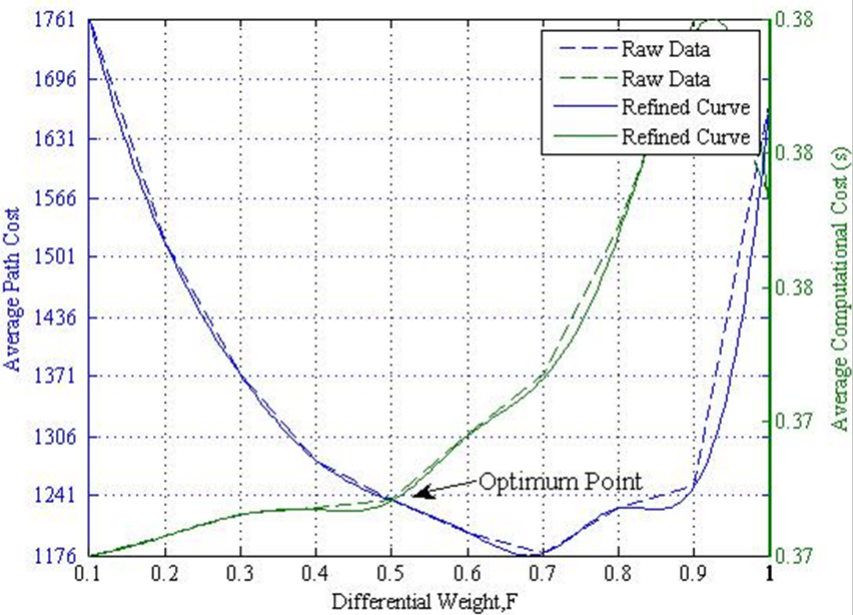
Estimation of optimum differential weight at NP = 10, G = 1000, and CR = 100%.

Fig 4 presents an example for obtaining the optimum differential weight at a population size of 10, generation number of 1000, and crossover rate of 100%. Each interception point from all combinations of crossover, population size, and generation number is recorded by using the same method. Hence, the optimum differential weight for different crossover rates at various population sizes from a generation number of 200 to 1000 is obtained.

**Fig 5:**
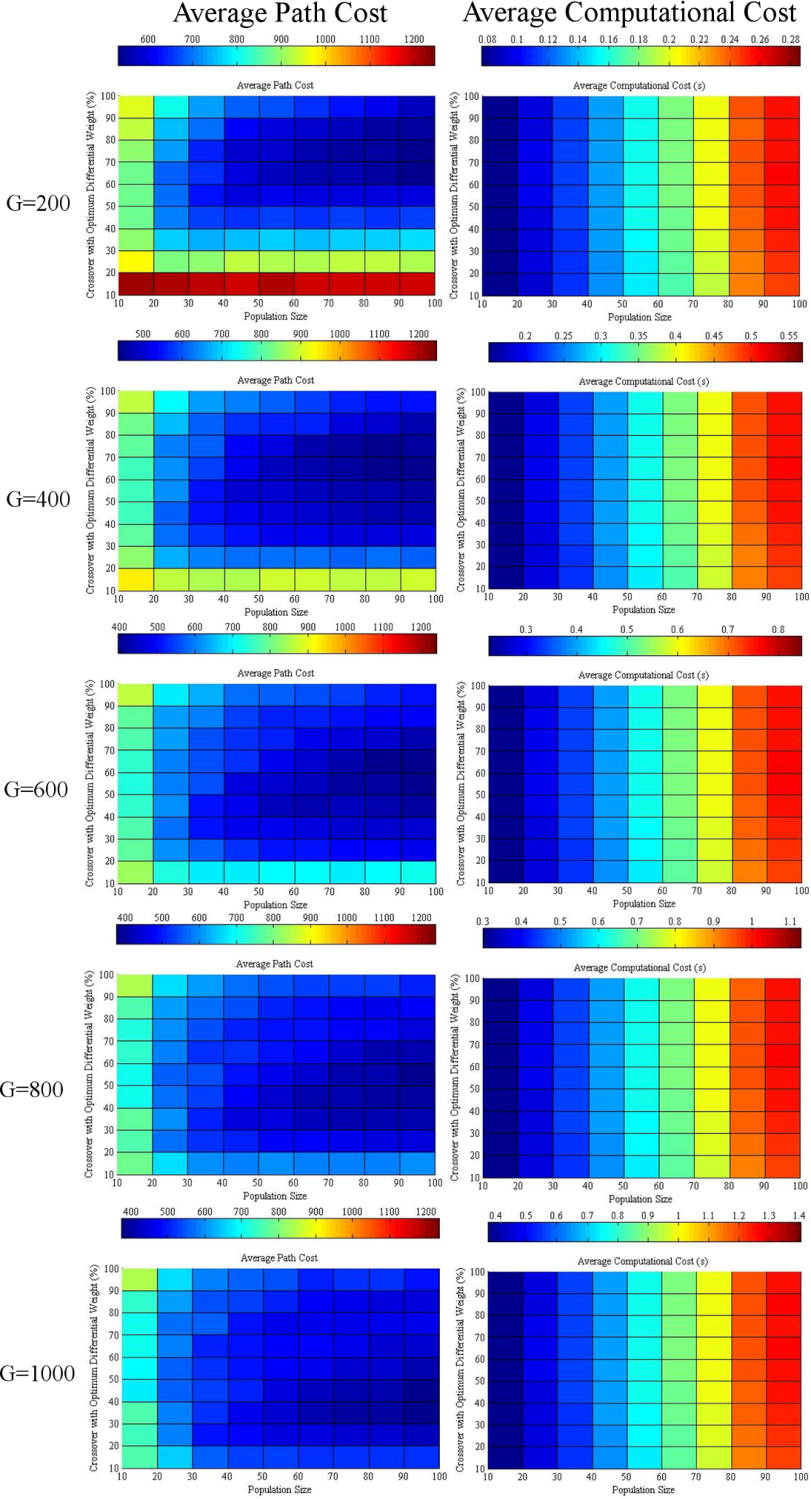
Average path and computational cost obtained with crossover rates on the optimum differential weight over population sizes at various generations of 200, 400, 600, 800, and 1000 “Table B in S1 Dataset.”

**Fig 6:**
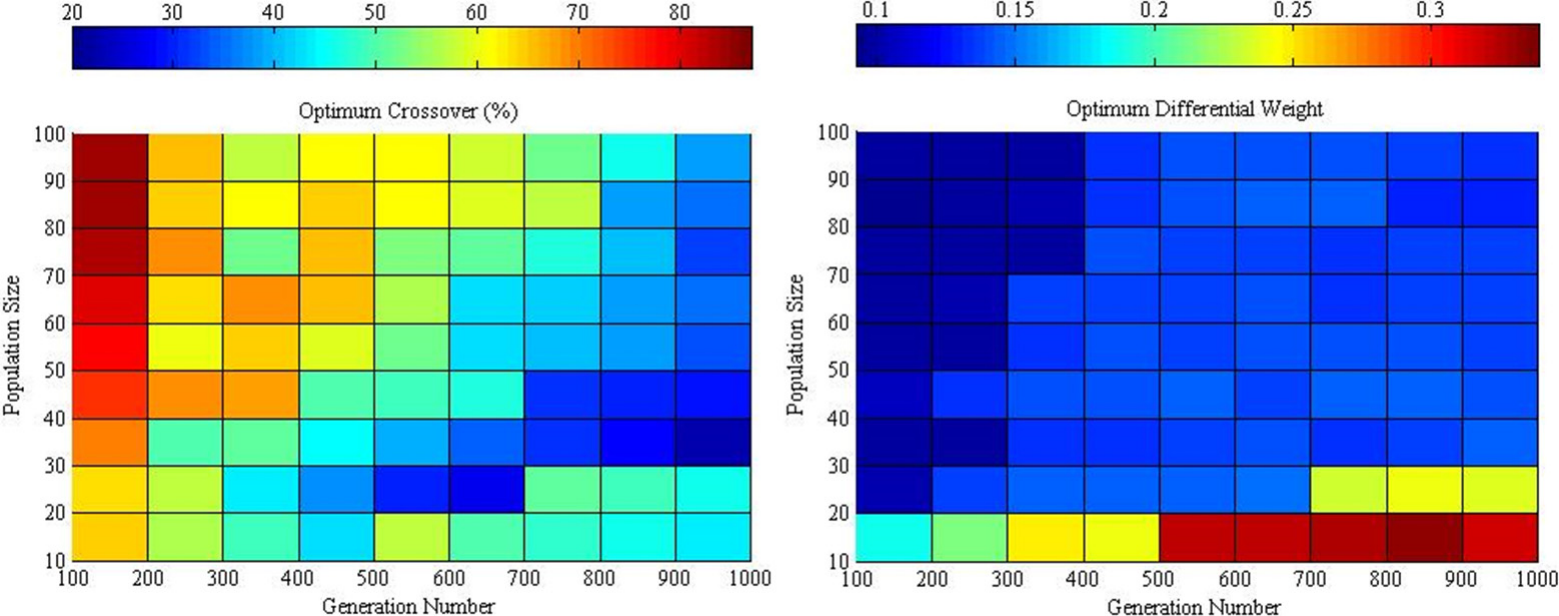
Optimum crossover and differential weight for various population sizes and generations “Table C in S1 Dataset.”

In each generation, the average computational cost of various crossovers for the same population size is almost the same for all cases in the same generation number, as given in Fig 5. Furthermore, the trend of the average path cost from various crossovers for the same population size is a parabola with a minimum point. Thus, we assume that the optimum crossover with its optimum differential weight can be determined at the lowest average path cost of a particular population size. Fig 6 displays the optimum crossover value and differential weight for different population sizes from a generation number of 100 to 1000.

### Overall Optimization of Control Parameters

**Fig 7:**
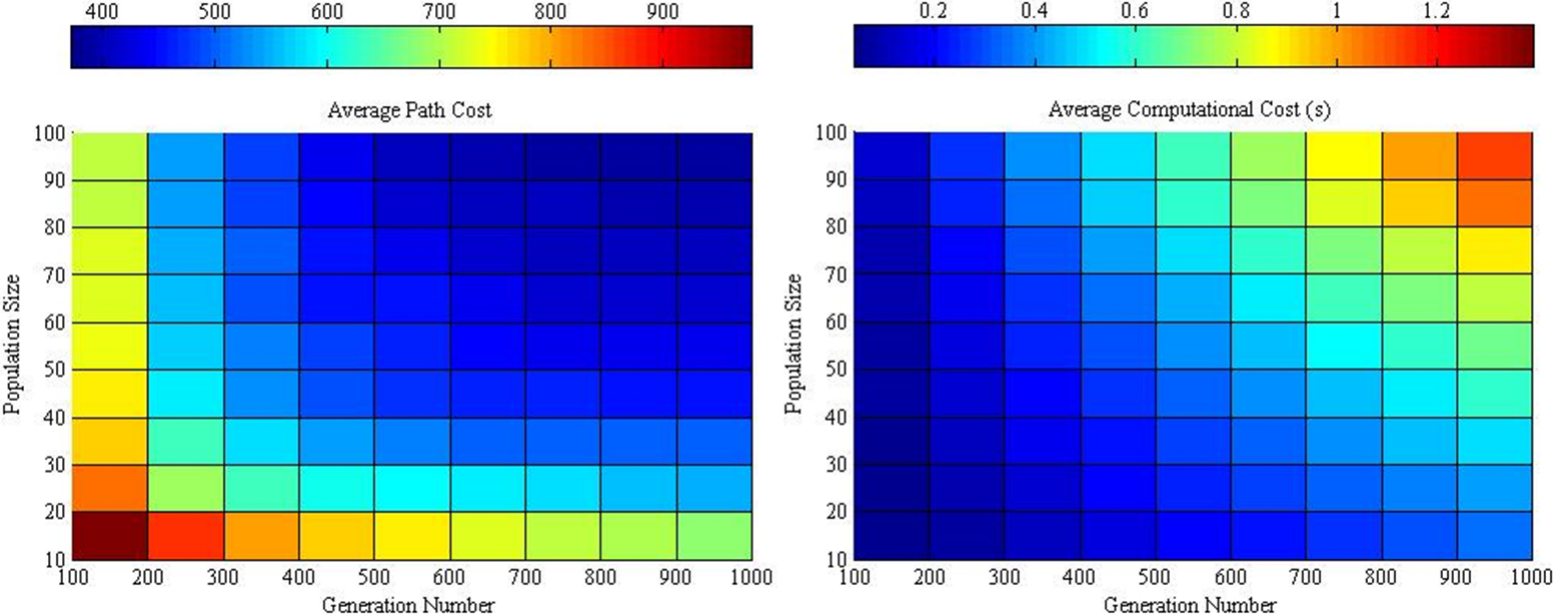
Average path and computational cost between various population sizes and generation numbers at the optimum crossover and differential weight “Table D in S1 Dataset.”

The optimum crossover has a tendency to decrease with increasing generation number, whereas the optimum differential weight is the opposite. A larger population size will have lower path cost and higher computational cost and vice versa (Fig 6). Moreover, a similar trend between the average path and computational cost is shown in Fig 7.

**Fig 8:**
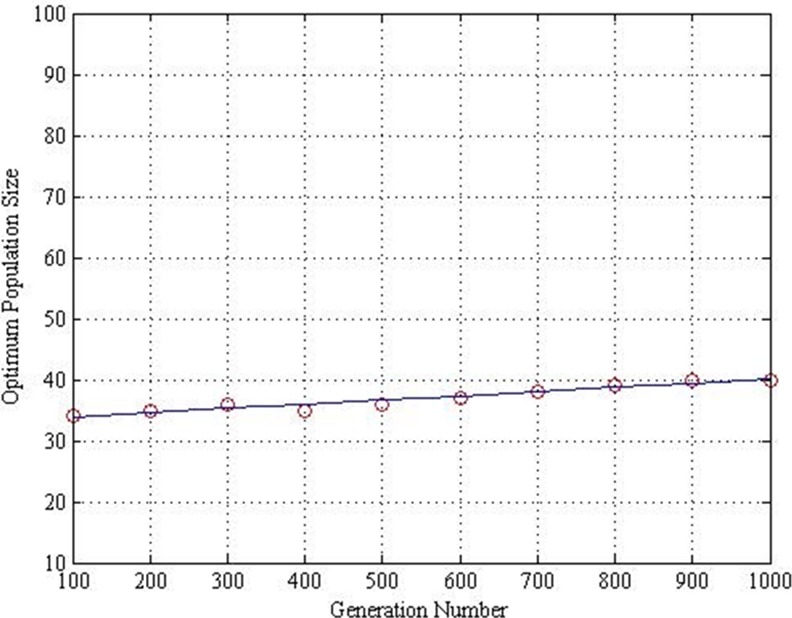
Optimum population size along generation number.

Thus, the interception point between both is identified as the balanced performance of the DE algorithm. The optimum population size at various generation numbers is illustrated in Fig 8. The figure indicates that the optimum population is almost consistent at the same level when the generation number increases within the range. Therefore, the optimum population size will not be affected evidently in these simulations from the initial stage to the convergence stage. The optimum population size in relation to the maximum generation numbers may be determined by using the following equation:

P=0.006909*G+33.2. (5)

**Fig 9:**
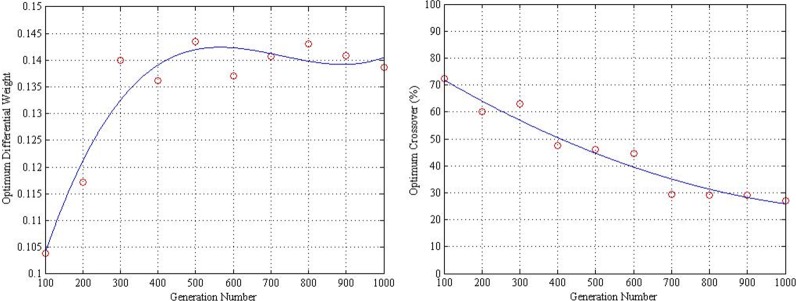
Optimum differential weight and crossover along generation number.

The optimum differential weight and crossover along the generation number is shown in Fig 9. At this instance, the number of generation is small when the optimum differential weight is small. As the generation number becomes larger, the optimum differential weight increases with decreasing rate. However, the optimum crossover decreases as the generation number grows with decreasing rate. Therefore, the optimum differential weight and crossover in relation to the generation numbers may be determined by using Eqs (6) and (7), respectively,

$$F=1.858\;*\;10^{-10}G^{3}-4.068\;*\;10^{-7}G^{2}+0.0002821G+0.07939$$ (6)

$$CR=80.55-8.864\;*\;10^{-2}G+3.374\;*\;10^{-5}G^{2}\;.$$ (7)

The optimized setting of population size, differential weight and crossover using Eqs (5) till (7) for different maximum generation number is shown in Table 1. This simplifies the need to trial and error of the crucial parameters especially population size, differential weight and crossover for the specific maximum generation number. As a result, the intended output to obtain the desired path distance and computational time may be achieved for aircraft path planning mission.

**Table 1:** Optimized setting of population size, differential weight & crossover at various maximum generation number.

Generation	100	200	300	400	500	600	700	800	900	1000
**Population**	34	35	35	36	37	37	38	39	39	40
**Differential Weight**	0.10	0.12	0.13	0.14	0.14	0.14	0.14	0.14	0.14	0.14
**Crossover (%)**	72	64	57	51	45	40	35	31	28	26

### Proposed DE Algorithm Optimization Result

**Fig 10:**
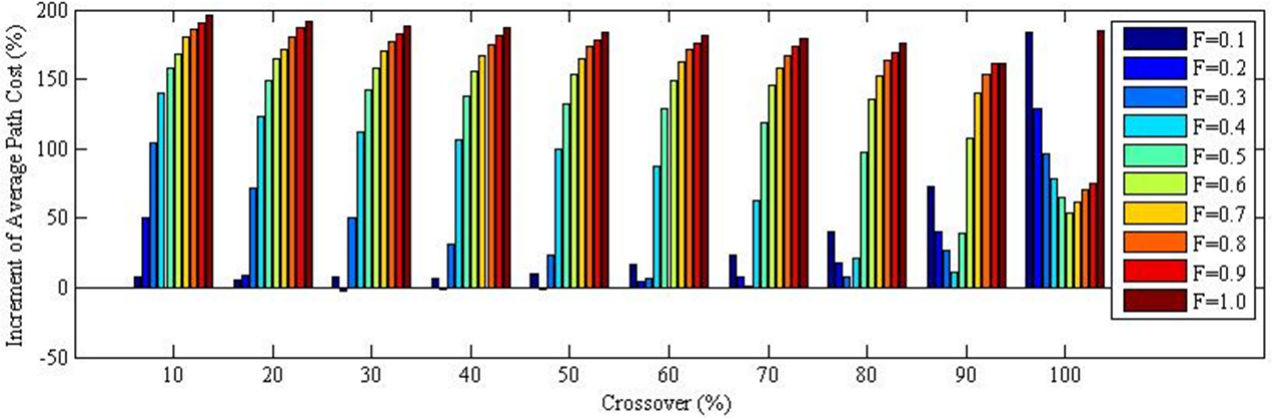
Average path cost changes in % when compared to the optimized parameter setting at maximum generation number of 1000 “Table E in S1 Dataset.”

The analyses on the effect of using and optimized DE algorithm are presented. In this occasion, a simulation was done for a maximum generation number input value of 1000, to make a performance comparison between the optimized parameter setting (i.e. shown in Table 1) and the all other setting. Fig 10 illustrates the average path cost changes in percentage when compared to the optimized parameter setting at maximum generation number of 1000.

These analyses clearly indicates that the average path cost at optimized parameter setting is better than all other setting, except for 3 combination setting (i.e. when Differential Weight is 0.2 and Crossover rate of 30, 40 and 50%). Still, the path cost at these non-optimized parameter setting are only better in the range of 1–3%. Moreover, the average path cost at all other setting are generally higher than optimized parameter setting, which may be by almost 200%.

**Fig 11:**
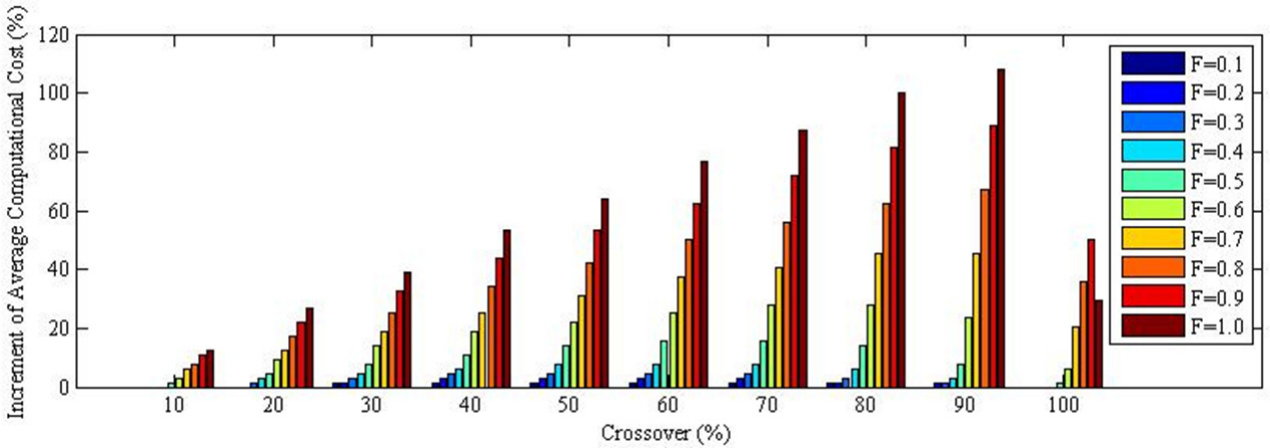
Average computational cost changes in % when compared to the optimized parameter setting at maximum generation number of 1000 “Table F in S1 Dataset.”

The average computational cost changes in percentage when compared to the optimized parameter setting at maximum generation number of 1000 is shown in Fig 11. In contrast to the path distance cost, the computational time of optimized parameter setting were better than all non-optimized parameter setting. In addition, some non-optimized parameter setting have achieved more than 100% increment in computational time. This clearly indicates the importance for an optimized parameter setting in DE algorithm for aircraft path planning using optimized setting based on maximum generation number.

## Conclusion

Instead trial and error, an optimization of the DE algorithm for tuning the parameters of UAV path planning is presented in this paper. The parameters focused are population size, differential weight, crossover, and generation number. In conclusion, the proposed optimization of the tuning parameters in the DE algorithm for UAV path planning has enabled an expedited and improved simulation of the final output path and computational cost. The developed algorithm enables the user to simply define the weightage desired between the path and computational cost to converge with the minimum generation required based on user requirement.

## Supporting Information

S1 Dataset Path and computational cost data of UAV path planning using DE. (XLSX)
